# Multi-omics study on the effect of moderate-intensity exercise on protein lactylation in mouse muscle tissue

**DOI:** 10.3389/fcell.2024.1472338

**Published:** 2025-01-28

**Authors:** Jiahui Chang, Wanyu Wu, Ping Qian, Zhaoxu Lu, Xuejia He, Fang Wang, Ting Zhang

**Affiliations:** ^1^ Children’s Hospital Capital Institute of Pediatrics, Chinese Academy of Medical Sciences and Peking Union Medical College, Beijing, China; ^2^ Beijing Municipal Key Laboratory of Child Development and Nutriomics, Capital Institute of Pediatrics, Beijing, China; ^3^ Graduate School of Peking Union Medical College, Beijing, China; ^4^ Department of Internet Medicine, Affiliated Children Hospital of Capital Institute of Pediatrics, Beijing, China; ^5^ Beijing Municipal Key Laboratory of Child Development and Nutriomics, Capital Institute of Pediatrics-Peking University Teaching Hospital, Beijing, China

**Keywords:** moderate-intensity exercise, lactylation, proteome, metabolome, muscle tissue

## Abstract

**Introduction:**

This study explores the effects of moderate-intensity exercise on protein lactylation in mouse muscle tissue metabolism.

**Methods:**

Healthy adult mice running for 6 weeks as an exercise model and sedentary mice as the control were used to perform transcriptomic, proteomic, lactylation-proteomic, and metabolomic analyses. Correlation analysis between transcriptome and proteome and between proteome and metabolome was also conducted.

**Results:**

In this study, 159 lactylation sites of 78 proteins were identified as being differentially regulated by moderate-intensity exercise. Enrichment analysis showed that the lactylation of proteins Atp5mg, and Atp5po exhibited ATP hydrolysis activity. Mtatp8 and Atp5po were involved in biological processes such as mitochondrial transmembrane transport, and Mtatp8, Atp5mg, and Atp5po participate in oxidative phosphorylation and thermogenesis pathways. The lactylation levels of Mtatp8, Atp5mg, and Atp5po proteins in the exercise group were significantly decreased, while their protein levels were significantly increased. The combined analysis of proteomics and metabolomics showed that the oxocarboxylic acid metabolism and sphingolipid signaling pathways had significant changes under the influence of moderate-intensity exercise.

**Discussion:**

Our results indicate that moderate-intensity exercise has an effect on the lactylation level of mice, possibly by reducing the lactylation levels of Mtatp8, Atp5mg, and Atp5po and increasing the expression of their protein levels, thereby regulating the oxidative phosphorylation pathway and participating in energy metabolism. Further exploration is needed into the 2-oxocarboxylic acid metabolism pathway and the sphingolipid signaling pathway.

## 1 Introduction

Scientific exercise is beneficial to human physical health and can mitigate the progression of various diseases, such as metabolic (obesity, diabetes, etc.), nervous system, and cardiovascular diseases (Zangger et al., 2023; Zhao, 2024). Muscles and bones are important parts of the body that mediate the impact of exercise on physical activity. They are also key endocrine organs and play an important role in resisting chronic inflammation and maintaining the body’s immune system function (Kaji, 2023; Yang D. et al., 2023). The influence of exercise on muscles is a complex and multifaceted adaptive response (Marusic et al., 2020). Exercise can cause physiological and morphological changes, such as muscle growth, increased strength, improved endurance, and metabolic changes in a variety of muscles(Tsekoura et al., 2021). At the same time, strenuous exercise can also lead to muscle damage, which requires proper repair (Clarkson and Hubal, 2002; Cheng et al., 2020). During exercise, muscles produce a large number of active factors and metabolites that are released to the target parts of the body to perform a variety of biological functions, mediating the regulatory effect of exercise on pathophysiological conditions through muscle tissue (Lee et al., 2023). Among these, lactic acid produced through exercise is closely related to energy metabolism – it is—used as a key marker of fat oxidation in the skeletal muscle and plays an important role in tissues and organs such as the heart, brain, kidney, and liver (Brooks, 2018; Yang et al., 2020). Lactate is used as a key indicator in research to assess the effects of exercise training on the body.

A novel protein post-translational modification—lactylation modification—was reported for the first time by Zhang et al. (2019), providing a new perspective for the study of the non-metabolic functions of lactic acid. Lactylation modification originates from lactate produced by cellular glucose metabolism and is regulated by glycolysis and mitochondrial oxidative metabolism. As an epigenetic regulator, lactic acid directly stimulates gene transcription in chromatin and regulates the expression of related genes through the epigenetic modification of histone lactylation (Chen et al., 2022). Lactate stimulates gene transcription by inducing histone lactylation (Kla) in M1 macrophages, disrupting the balance of transcription (Zhang et al., 2019). The progression of diseases such as tumors and inflammation promotes the entry of lactic acid into macrophages (Fan et al., 2023). Histone lactylation levels are elevated in tumors and are closely associated with poor prognosis and epigenomic reprogramming (Yu et al., 2021). In addition to the acylation of histone proteins, lactate can also mediate the acylation of non-histone proteins that are involved in key cellular processes relevant to physiology and disease (Gao et al., 2023; Yang Z. et al., 2023).

Research has shown that the function of lactylation-modified regulatory proteins involves two pathways (Xin et al., 2022). ① Kla has a high content in the gene promoter region and can directly bind to the promoter region to promote or inhibit the expression of certain genes at the transcriptional level. ② Lactylation directly modifies the protein and regulates its activity. However, it is largely unknown how the overall lactylation levels change in muscle tissue during exercise and the specific pathway of action of lactylated proteins.

In our study, transcriptomic, proteomic, lactylation-proteomic, and metabolomic sequencing were performed based on mouse muscle tissue samples from moderate-intensity exercise to clarify the lactylation effects of moderate-intensity training on muscle tissue. The impact of acylated modified proteins can elucidate the role of lactylated proteins as a bridge between energy metabolism and epigenetics. This study has deepened our understanding of the specific physiological mechanisms of exercise’s regulation of muscle tissue.

## 2 Materials and methods

### 2.1 Moderate-intensity exercise training protocol

Our study selected 8-week-old male C57BL/6J mice (weighing approximately 21 ± 1 g, purchased from Charles River Laboratory) and randomly divided them into a control (Con) and exercise (EX) group. The experiment strictly followed institutional ethical guidelines and was approved by the Animal Care and Use Ethics Committee of the Capital Institute of Pediatrics (DWLL2021015) of Beijing, China. The EX group trained according to a moderate-intensity exercise program (Qian et al., 2022; Lu et al., 2023)—the mice trained on the electric treadmill between 7:00 and 9:00 pm, and adapted to the treadmill for 10–15 min every day for 5 days. The speed started at 6 m/min, followed by 2 m/min acceleration until the final velocity per day (Supplementary Table 1). In the training phase, EX mice started running at 6 m/min per time and increased to the specified speed, 60 min per day, 5 consecutive days per week, for a total of 6 weeks of training; the running distance of each mouse was 22 km. The Con mice were not treadmill trained and were fed according to feeding conditions throughout. All mice were kept at a constant temperature (22 °C ± 2 °C), with a diurnal cycle (light turned on at 6:00 am., and a night phase at 6:00 pm, maintaining a 12-/12-h light/dark cycle) and were weighed every 2 weeks.

### 2.2 Tissue collection

The mice in the EX group were euthanized and killed by cervical dislocation 24 h after the last training session. We used phosphate-buffered saline (PBS) for transcardial perfusion, and the cadavers were disinfected with 70% ethanol and placed on a dissection table with the abdomen facing up. The hind leg muscles were removed and placed in the prepared Petri dish, and intermuscular fat was removed (Hastings et al., 2020). The tissue samples were packaged and stored according to the requirements of each omics sequence: the size of soybean grains was required for transcription, and the amount of tissue was required to reach 50–100 mg for metabolomics, >200 mg for proteomics, and >500 mg for modified proteomics. The collected tissue samples were quickly frozen in liquid nitrogen and transferred to −80°C for subsequent trials.

### 2.3 Nucleic acid RNA extraction and transcriptomic sequencing analysis

First, TRIzol reagent was used to extract total RNA from muscle tissue samples and purify it to ensure that the quality and concentration of the RNA samples met the requirements of subsequent sequencing. A library was constructed and sequenced based on the DNBseq platform (PE150 strategy). Low-quality sequences, such as head sequence, sequence with mass score <20, original reads with N-base ratio >10%, and sequence less than 25 bp, were removed from the RNA sequencing results. The clean reads thus obtained were matched to the reference genome GCF_000001635.27_GRCm39 using HISAT. The expression quantification of the data was conducted, the differential expression gene analysis was conducted based on DESeq, and functional annotation and concentration analysis were conducted for the Gene Ontology (GO) and Kyoto Encyclopedia of Genes and Genomes (KEGG) databases (Lu et al., 2023).

### 2.4 Protein extraction and enrichment of lactylated peptides

The muscle tissue samples frozen with liquid nitrogen were ground to powder. Subsequently, a cracking buffer (8 M urea, 1% protease inhibitor, 3 μM trichostatin A (TSA), and 50 mM nicotinamide (NAM)), with a volume four times that of the powder, was added. After adding the buffer, ultrasonic cracking was performed. The resulting mixture was centrifuged at 4 °C, 12,000 g for 10 min, and then the cell debris was removed. We transferred the supernatant to a new centrifuge tube and determined the protein concentration by the BCA kit.

The same amount of protein was taken from each sample for enzymatic hydrolysis. The volume was adjusted to the same with the lysate, and then trichloroacetic acid (TCA) was slowly added to reach a final concentration of 20%. The solution was thoroughly mixed by vortexing and allowed to precipitate at 4°C for 2 h. Subsequently, it was centrifuged at 4,500g for 5 min, the supernatant was discarded, and washed the precipitate with pre-cooled acetone two to three times. After drying the precipitate, triethylammonium bicarbonate buffer (TEAB) was added to a final concentration of 200 mM, the precipitate was dispersed by ultrasound, trypsin was added at a ratio of 1:50 (protease: protein m/m), and enzymatic hydrolysis was carried out overnight. Dithiothreitol (DTT) was added to a final concentration of 5 mM and reduced at 56 °C for 30 min. Iodoacetamide (IAA) was then added to make a final concentration of 11 mM and incubated at room temperature for 15 min in the absence of light. The peptides obtained were desalted and vacuum-dried for subsequent proteomic sequencing.

The process of protein extraction and enzymolysis for lactylated protein modification was the same as above, and the peptides were dissolved in IP buffer solution (100 mM NaCl, 1 mM EDTA, 50 mM Tris-HCl, 0.5% NP-40, pH 8.0). We transferred the supernatant to the pre-washed resin (No. PTM1404) and placed it on a rocking shaker overnight at 4 °C for incubation. Subsequent washes were performed using IP buffer solution, deionized water, and 0.1% trifluoroacetic acid eluent, respectively. We collected and drained the eluent by vacuum freezing. After draining, it was desalted according to the C18 ZipTips instructions, then vacuum frozen and drained for liquid chromatography/ mass spectrometry (LC/MC) analysis.

### 2.5 Sequencing and analysis of proteomics and lactylated modification

Detection data of proteomics and lactylated modification were obtained by LC/MC. The obtained peptides were dissolved by liquid chromatography mobile phase A and separated using the NanoElute ultra-high performance liquid phase system. Mobile phase A is an aqueous solution containing 0.1% formic acid and 2.0% acetonitrile. Mobile phase B is an acetonitrile-aqueous solution containing 0.1% formic acid. The liquid phase gradient setting (proteome setting condition) was as follows: 0–70 min, 6–24%B; 70–82 min, 24–35%B; 82–86 min, 35–80%B; 86–90 min, 80%B. The liquid phase gradient setting of the lactylated group was as follows: 0–40 min, 7–24%B; 40–52 min, 24–32%B; 52–56 min, 32–80%B; 56–60 min, 80%B. The separated peptides were injected into the capillary ion source for ionization and then analyzed by timsTOF Pro mass spectrometry. The source voltage of the proteome was set to 1.75 kV, and the source voltage of the lactoacylated group was set to 1.7 kV. The dynamic exclusion time of the tandem mass spectrometry scan was set to 30 s for the proteome (24 s for the lactoacylation modification group) to avoid repeated scanning of parent ions. The MaxQuant search engine (v. 1.6.15.0) process was generated from MS/MS data. The tandem mass spectrum was searched against Mus_musculus_10090_SP_20220107.fasta (17,097 entries) connected to the reverse bait database. The false discovery rate (FDR) for protein, peptide, and acetyl sites was adjusted to <1% (Qian et al., 2022).

In quantitative omics studies, the relative signal intensity ratio (Ratio) of the protein or modification site between the experimental and control groups is used to measure the difference in its expression level under two states. To ensure that the expression difference was statistically significant, the difference significance test T-test *p* value was calculated by repeating the experimental results several (three or more) times.

### 2.6 Metabolite extraction and omics sequencing analysis

Samples were collected at −80°C, and appropriate tissue samples were weighed into a mortar pre-cooled with liquid nitrogen and fully ground to powder. Then, four times the volume of extraction buffer MeOH/ACN (1:1, v/v) was added to each group of samples, and ultrasonic cracking was performed after full vorticity homogenization. It was quickly frozen in liquid nitrogen for 1 min and thawed at room temperature, and then ultrasonic treatment was performed again. We repeated this step three times. After precipitation at −20 °C for 1 h and centrifugation at 4 °C at 18,000 g for 15 min, the cell debris and protein precipitate were removed, the supernatant was transferred to a new centrifuge tube, drained through a concentrator, and ultrasonically redissolved with equal volumes of ACN: H2O (1:1, v/v) added. Centrifuged at 4 °C, 18,000 g for 15 min, the supernatant was transferred to a new centrifuge tube and stored at −80 °C or LC/MS for computer analysis.

The metabolites were separated using the Waters UPLC ultra-high performance liquid phase system combined with the Waters ACQUITY UPLC BEH C18 Column (1.7 µm, 2.1 mm × 100 mm), with a sample size of 10 µL and elution at a flow rate of 400 µL/min, column temperature of 40 °C. Mobile phase A was an aqueous solution containing 0.1% formic acid, and mobile phase B was acetonitrile containing 0.1% formic acid. Liquid phase gradient setting: 0–11 min, 2%∼98%B; 11.0–12.0 min, 98%B; 12.0–12.1 min, 98%∼2%B; 12.1–15.0 min, 2%B. The separated metabolites were injected into the ESI ion source for ionization and then analyzed by timsTOF Pro mass spectrometry. The ion source voltage was set to 4.5 kV. The dynamic exclusion time of the series mass spectrometry scan was set to 6 s to avoid the repeated scanning of parent ions.

Based on the quantitative information of the metabolites obtained through database matching, data screening, and statistical algorithms were combined to fill in and correct the missing values of the data. For the samples with multiple repetitions, the corrected expression level was used to calculate the metabolite difference fold change (FC) between the two groups, and the P value of the univariate T-test analysis was combined. Multivariate statistical analysis orthogonal partial least square discriminant analysis (OPLS-DA) calculated variable importance in projection (VIP) value and further obtained significant difference metabolites.

### 2.7 Nine-quadrant diagram analysis

Through the association analysis of the two omics, the key protein sites related to the studied traits could be found, and the number of candidate protein sites could be reduced, which made the study more convenient. A nine-quadrant diagram was used to correlate the changes in loci and proteins. All protein sites were divided into nine regions according to two omics thresholds, with the *X*-axis representing the logFC value of the site in the proteome and the *Y*-axis representing the log2FC value of the site in the modified group. Different colors represent different modes of expression.

### 2.8 Real-time quantitative polymerase chain reaction (RT-qPCR)

Total RNA was extracted from tissue homogenates using TRIzol reagent (Invitrogen, United States) following the manufacturer’s protocol. Reverse transcription of RNA into cDNA was carried out using the RevertAid First-Strand cDNA Synthesis Kit (ABM, Canada). Real-time quantitative PCR (RT-qPCR) was conducted with Maxima SYBR Green/ROX qPCR Master Mix (ABM, Canada). The threshold cycle (Ct) values for each sample were determined, and relative expression levels were calculated using the 2^−△△Ct^ method, normalized to GAPDH as the reference gene. The primer sequences used in the analysis are provided in Supplementary Table 2.

### 2.9 Western blot (WB)

The membranes were incubated overnight at 4 °C with the following primary antibodies: MT-ATP8 (1:2,000, Proteintech, China), ATP5MG (1:2,000, Proteintech, China), ATP5PO (1:2000, Proteintech, China), and Anti-L-Lactyl Lysine (1:2,000, PTM Bio, China). GAPDH (1:6,000, Abcam, United Kingdom) was used as the loading control. After primary antibody incubation, the membranes were washed and then incubated at room temperature for 1 h with a secondary anti-rabbit antibody (1:9,000, CST, United States). The target protein bands were visualized using SuperSignal West Pico chemiluminescence substrate (Thermo Fisher Scientific, United States), and band intensities were quantified using Quantity One software (Bio-Rad Universal Hood II, United States).

## 3 Results

### 3.1 Identification of the lactylation landscape in mouse muscle tissue

**FIGURE 1:**
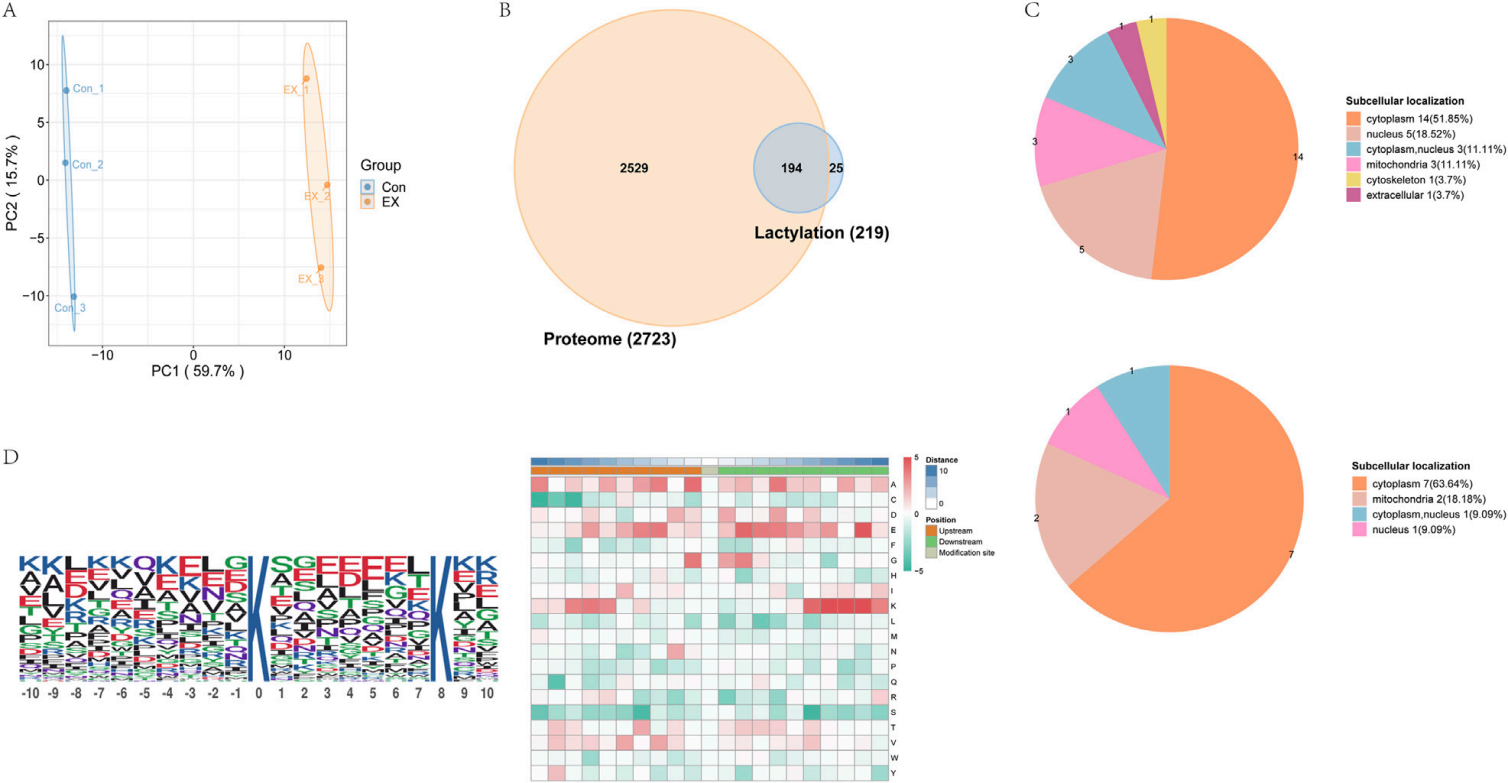
Identification of the lactylation landscape in mouse muscle tissue. **(A)** Principal component analysis of lactylation data to distinguish between EX and Con. **(B)** Venn diagram comparing lactylation group and proteome identification . **(C)** Number of differentially lactylated proteins (top-down sites and bottom-up sites) in different subcellular structural species. **(D)** WebLogo consensus map (left) and motifs of modification sites analyzed by MoMo (right).

We evaluated lysine lactylation (Kla) enrichment in muscle tissue from moderate-intensity training mice using LC-MS/MS analysis. The relative quantitative value of the modification site was divided by the relative quantitative value of the corresponding protein at the modification site to eliminate the influence of protein expression on the modification expression (Supplementary Table 3). Principal component analysis of the relative quantitative values of the samples showed a high degree of repeatability of the samples within the group (Figure 1A). We identified 915 acetyl sites on 219 proteins (Figure 1B, Supplementary Figure S1). The six proteins with the most acetyl sites were Ttn (170 sites), Myh4 (100 sites), Myh1 (26 sites), Ckm (19 sites), Mybpc2 (16 sites), and Tpm (14 sites). In addition, 149 proteins at 667 lactylation sites were quantified, and their distribution in each sub-cell was predicted (Supplementary Figure S1), with the most prominent locations being the cytoplasm, mitochondria, and nucleus (Figure 1C). The motif-X-based MoMo assay tool was used to understand the lactylation modification preferences of peptide sequences consisting of 10 amino acids—that is, −10 to +10 for all identified Kla sites. Based on the analysis results, the amino acid sequences around the lactylation sites were displayed in the heatmaps to determine the flanking sequence of the Kla site (Figure 1D). Finally, a conserved amino acid sequence was extracted and displayed in the WebLogo consensus map (Figure 1D left). The results showed that some amino acid residues around the Kla site were significantly enriched. Residues K were enriched at +6 to +9 and −8 to −6 (Figure 1D right).

### 3.2 Functional enrichment of differential lactylation-related proteins

**FIGURE 2:**
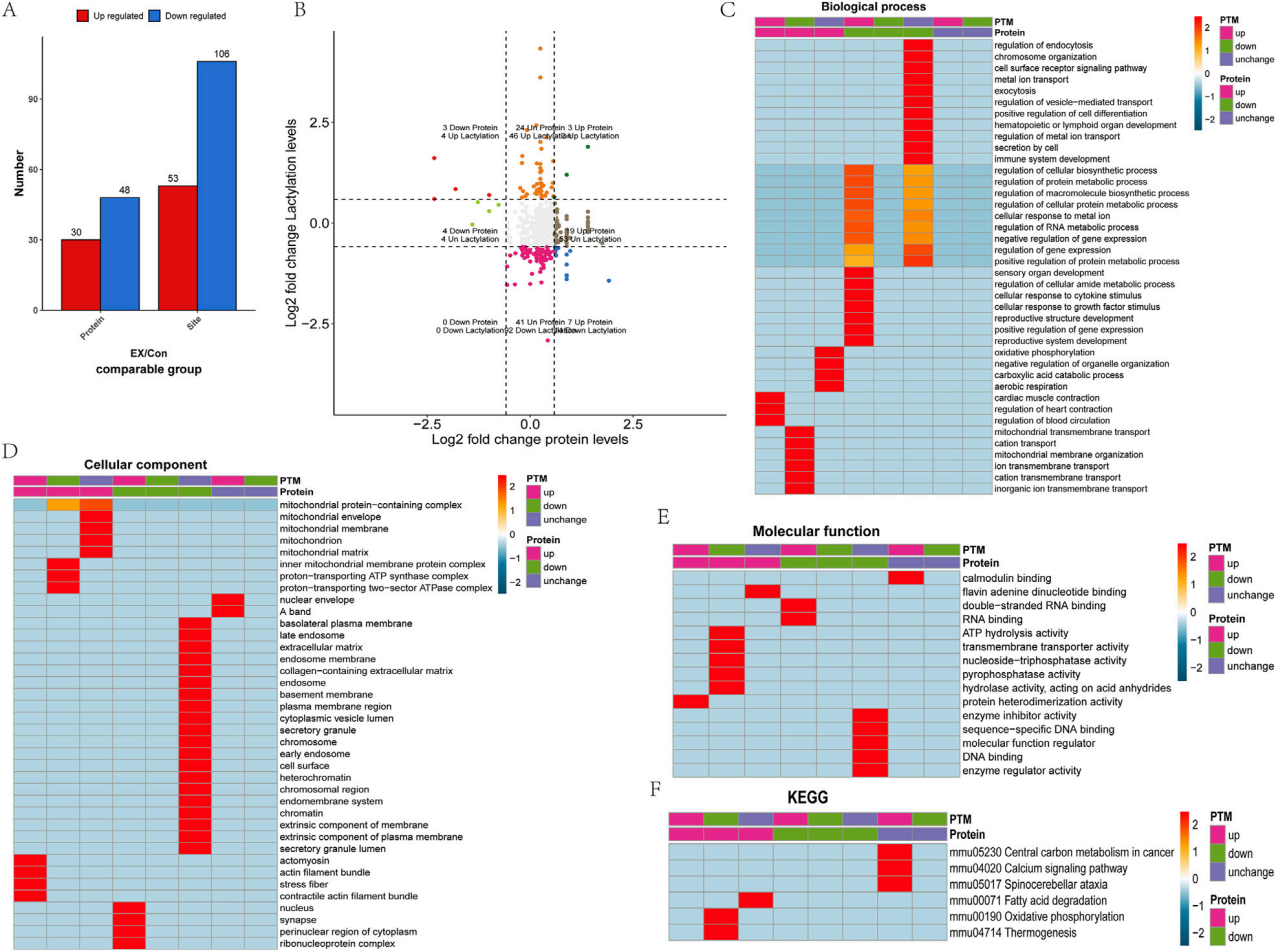
Functional enrichment of differential lactylation-related proteins. **(A)** Statistical analysis of differentially lactylated sites and proteins. **(B)** Comparison of differentially expressed proteins between the lactylation group and the proteome analyzed in the nine quadrant diagram analysis. **(C)** Functional analysis of the biological processes of different lactylation proteins. **(D)** Functional analysis of the cellular component of different lactylation proteins. **(E)** Functional analysis of the molecular function of different lactylation proteins. **(F)** KEGG analysis of the pathway of different lactylation proteins.

According to the differential screening criteria of proteins and lactylation modification sites, FC > 1.5 or FC < 1/1.5 significantly up- and downregulated differential proteins and differential modification sites were screened. The results showed a total of 78 differential proteins and 159 differential modification sites, including 30 upregulated proteins, 53 upregulated lactylation sites, 48 downregulated proteins, and 106 downregulated lactylation sites (Figure 2A). The nine-quadrant diagrams showed the lactylation modification site and protein changes in the comparison between the two groups (Figure 2B). The results showed that the lactylation modification level was higher than the protein expression level in quadrants 1, 2, and 4. Quadrants 3 and 7 indicated that lactylation modification was consistent with the regulation of protein expression levels. Quadrants 6, 8, and 9 indicated that the modification level was lower than the protein expression level. Among them, quadrants 1, 3, 7, and 9 had significant regulatory characteristics, and a total of 13 differential proteins and 21 differentially modified sites were significantly correlated and regulated. Functional enrichment analysis of the proteins showed that Fabp4, Vim, and Hnrnpa1 (upregulated proteins at the one-quadrant modification sites were downregulated) were involved in the regulation of the RNA metabolic process, macromolecule biosynthetic process, cellular protein metabolic process, protein metabolism process, cellular biosynthetic process, and gene expression regulation and other biological processes (Figure 2C). Fabp4, Vim, and Hnrnpa1 were found to be cellular components of nuclear cells and to have an RNA-binding molecular function (Figures 2D, E). Tpm1 and Myh1 (the three quadrant upregulated sites corresponding to upregulated proteins) were found to be involved in regulating biological processes such as cardiac muscle contraction and blood circulation (Figure 2C). Tpm1 and Myh1 were found to be the cellular components of the actin filament bundle, stress fiber, and actomyosin (Figure 2D). At the same time, Tpm1 and Myh1 were shown to have molecular functions of protein heterodimerization activity (Figure 2E). Mtatp8, Atp5mg, and Atp5po (nine-quadrant sites were downregulated and the protein was upregulated) were shown to participate in ion transmembrane transport. Mtatp8 and Atp5po were also found to be involved in biological processes such as mitochondrial membrane organization and mitochondrial transmembrane transport (Figure 2C). Mtatp8, Atp5mg, and Atp5po were shown to be cellular components of the proton-transporting ATP synthase complex, the proton-transporting two-sector ATPase complex, the mitochondrial membrane protein complex, and the mitochondrial protein-containing complex and found to be involved in the molecular function of transmembrane transporter activity (Figures 2D, E). Atp5mg, Atp5po, and Myh1 were found to have molecular functions of ATP hydrolysis activity, pyrophosphatase activity, hydrolase activity, and nucleoside-triphosphatase activity. Mtatp8, Atp5mg, and Atp5po are involved in oxidative phosphorylation and thermogenesis pathways (Figure 2F).

### 3.3 Effects of moderate-intensity exercise on gene transcriptional levels in muscle tissue

**FIGURE 3:**
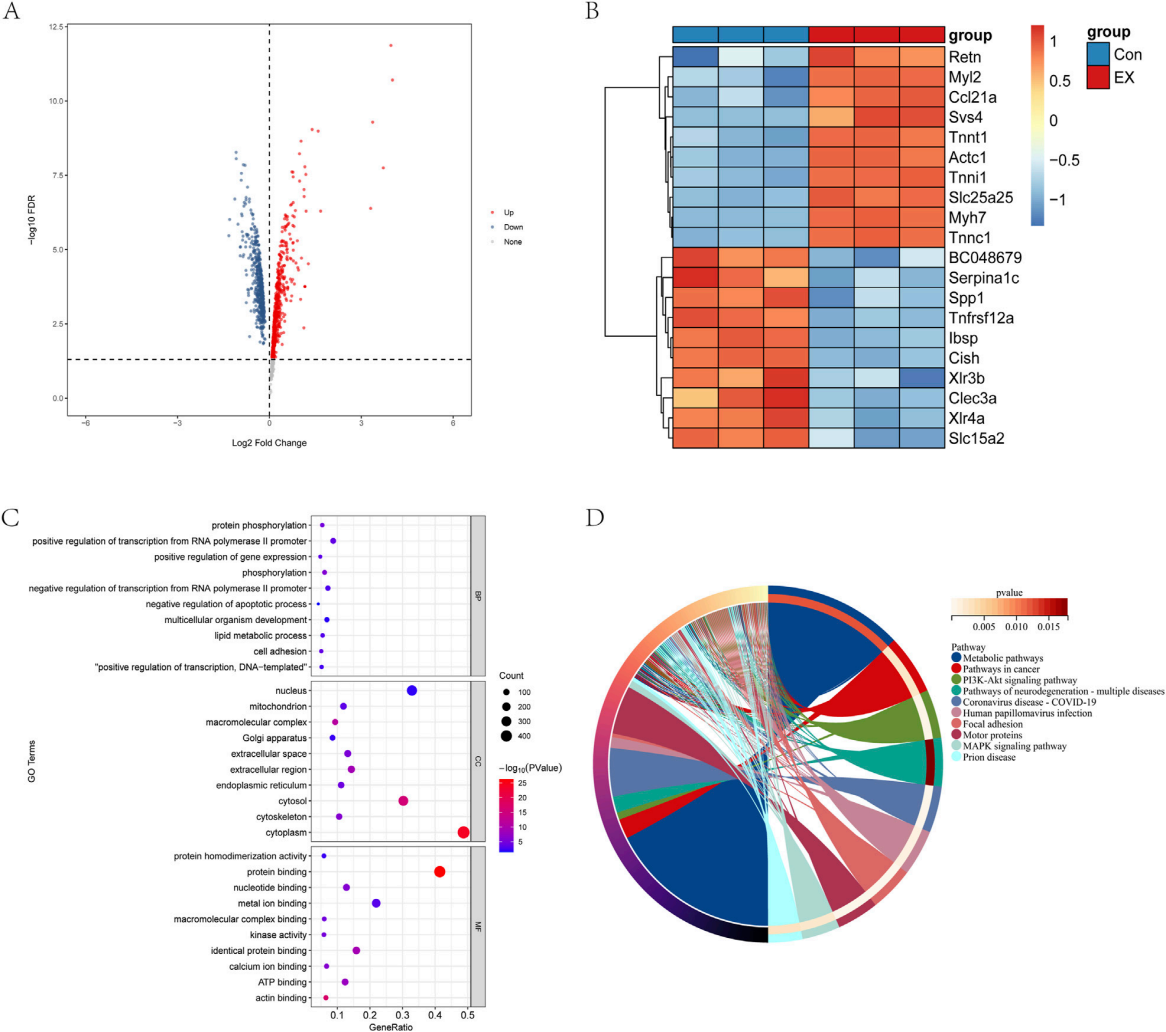
Effects of moderate-intensity exercise on gene transcriptional levels in muscle tissue. **(A)** Differential gene volcano map. **(B)** Heat map analysis of the top-10 significantly upregulated and top-10 significantly downregulated genes. **(C)** GO analysis of differential gene. **(D)** KEGG analysis of differential genes.

According to the screening conditions of gene differences, p value < 0.05 after verification and Log2 FC > 0 or Log2 FC < 0 were significant differentially identified genes, and a total of 497 significantly downregulated genes and 594 significantly upregulated genes were identified (Figure 3A). The heat map showed that the top ten differential genes were significantly upregulated and downregulated (Figure 3B). The differential genes were involved in biological processes such as the lipid metabolic process, cell adhesion, transcription regulation, and protein phosphorylation. Meanwhile, these differential genes were the cellular components of the cytoskeleton, nucleus, mitochondrion, Golgi apparatus, and endoplasmic reticulum, and they had the molecular functions of actin binding, ATP binding, metal ion binding, and protein binding (Figure 3C). However, the differential genes were mainly involved in metabolic pathways, PI3K-Akt signaling pathway, focal adhesion, and motor proteins (Figure 3D). The above results showed that the impact of moderate-intensity exercise on muscle tissue was mainly concentrated on energy metabolism, material transport, and other functions.

### 3.4 Correlation analysis of combined transcriptional levels of protein lactylation modification

**FIGURE 4:**
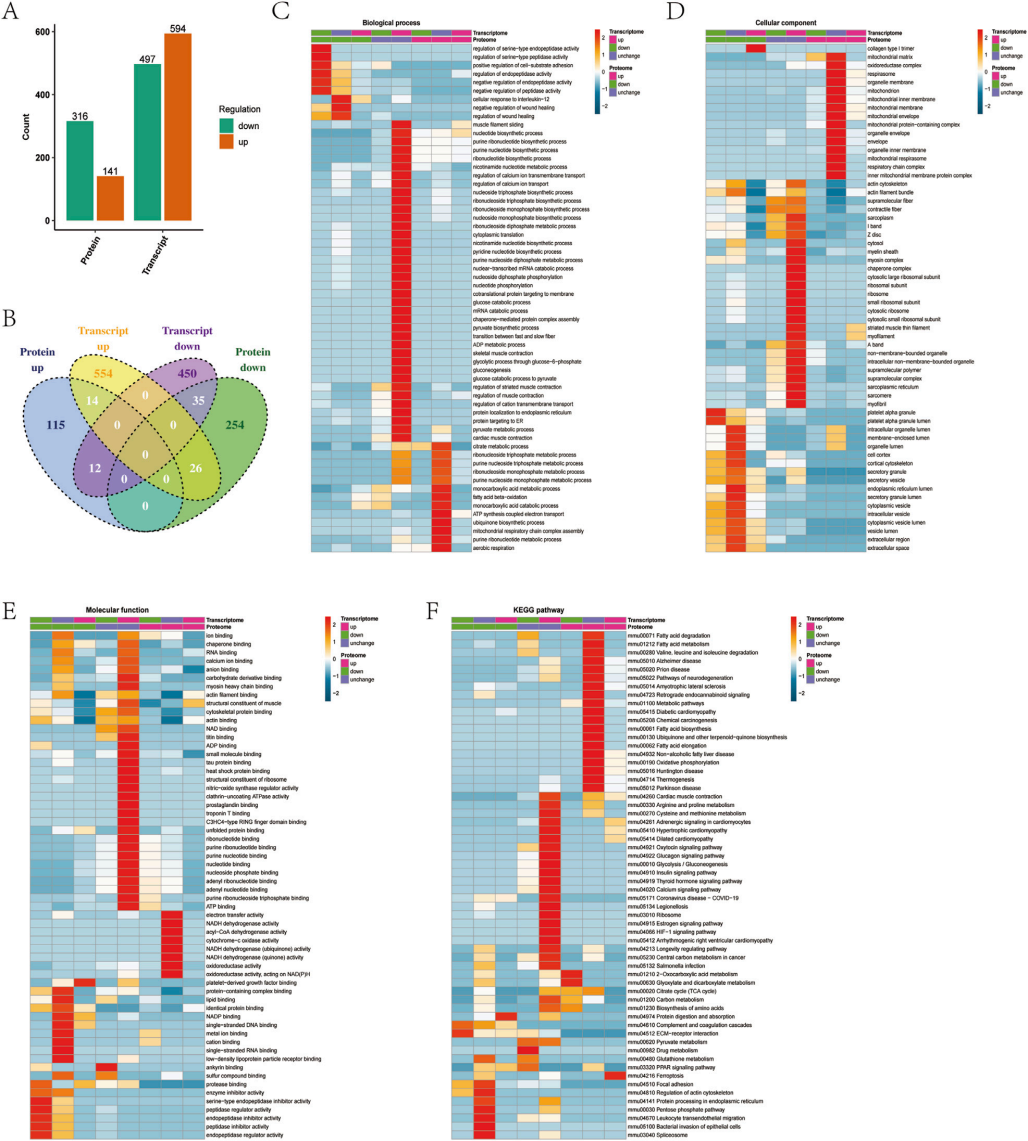
Correlation analysis of combined transcriptional levels of protein lactylation modification. **(A)** Statistical analysis of the difference of transcription and proteins. **(B)** Venn diagram for comparative analysis of differentially expressed proteins and transcription. **(C)** Functional analysis of the biological processes at both the transcriptional and protein levels. **(D)** Functional analysis of the cellular component at both the transcriptional and protein levels. **(E)** Functional analysis of the molecular function both at the transcriptional and protein levels. **(F)** KEGG analysis of the pathway at both the transcription and protein levels.

Transcriptomes and proteomes address the expression of genes at the transcriptional and translational levels, respectively. In order to determine whether factors regulated at the protein level overlap with those identified by transcriptional analysis, quantitative comparisons of transcriptomes and proteomes found 2,575 genes quantified at both transcriptomic and proteomic levels (Supplementary Figure 2A). By comparing the quantitative correlations between the two omics, we quickly understood the underlying regulatory relationship between proteins and transcripts. Since the amount of differentially expressed protein data is relatively small compared with the transcriptome, further FDR verification of the *p* value was not necessary. When the ratio >1.5 and the *p* value < 0.05, the expression of the upregulated protein was significantly different. When the ratio <1/1.5 and the *p* value < 0.05, the expression of downregulated protein was significantly different. The analysis resulted in 1,091 differentially expressed transcripts and 457 differentially expressed proteins, of which 594 were upregulated transcripts, 141 were upregulated proteins, 497 were downregulated transcripts, and 316 were downregulated proteins (Figure 4A). Among these, 49 proteins and transcripts had the same direction of action, of which 14 differentially expressed proteins and differentially expressed transcripts were significantly upregulated, and 35 differentially expressed proteins and differentially expressed transcripts were significantly downregulated (Figure 4B).

The upregulated genes and proteins Acsl3 and Pdhx were found to be involved in ribonucleotide biosynthetic and monocarboxylic acid metabolic processes, Tpm1 and Tpm3 were found to participate in biological processes such as muscle filament sliding, and Tpm1 and Mybph were found to participate in biological processes such as the regulation of muscle contraction (Figure 4C). Cryab, Tpm1, Tpm3, and Mybph were found to be the cellular components of muscle thin filament tropomyosin, actin cytoskeleton, and supramolecular, Pdhx and Ndufc2 were found to be the cellular components of oxidoreductase complex, and Acsl3 was found to be the cellular component of mitochondrial membrane (Figure 4D). Cryab, Tpm1, Tpm3, and Mybph were shown to have molecular functions of structural constituent of muscle, actin binding, cytoskeletal protein binding, and protein binding, and Acsl3 and Cryab were shown to have molecular functions of protein kinase binding (Figure 4E). KEGG analysis showed that Acsl3, Ndufc2, Shmt1, Pdhx, and Cycs participated in metabolic pathways, Tpm1 and Tpm3 participated in cardiac muscle contraction and adrenergic signaling in cardiomyocytes, and Ndufc2 and Cycs participated in oxidative damage phosphorylation (Figure 4F).

Eight proteins related to lactylation modification—Aco2, Fabp, Hnrnpa1, Mpc2, Mybph, Myh1, Tpm1, and Vim—were quantified at the transcriptional level (Supplementary Figure 2B). Among these, Mybph and Tpm1 were consistent at both transcriptional and protein expression levels and were significantly upregulated in exercise muscle tissue. Aco2 and Myh1 were downregulated at the transcriptional level and upregulated at the protein level. The phenomenon of inconsistent protein and transcription was found in other studies. Among them, Aco2 found to participate in the citrate metabolic process, tricarboxylic acid metabolic process, and aerobic respiration, and Myh1 found to participate in biological processes such as the ribonucleotide metabolic process. Aco2 was found to be a cellular component of the mitochondrion and mitochondrial matrix, and Myh1 was found to be a cellular component of stress fiber. Myh1 was found to have ATP binding, Aco2 was found to have molecular functions such as nucleoside phosphate binding and ribonucleotide binding, and Aco2 was found to have molecular functions such as transition metal ion binding and metal ion binding (Figures 4C–E). KEGG analysis showed that Idh3g, Aco2, and Cps1 were involved in the biosynthesis of amino acids, 2-oxocarboxylic acid metabolism, and metabolic pathways (Figure 4F).

### 3.5 Effects of moderate-intensity exercise on muscle tissue metabolite levels

**FIGURE 5:**
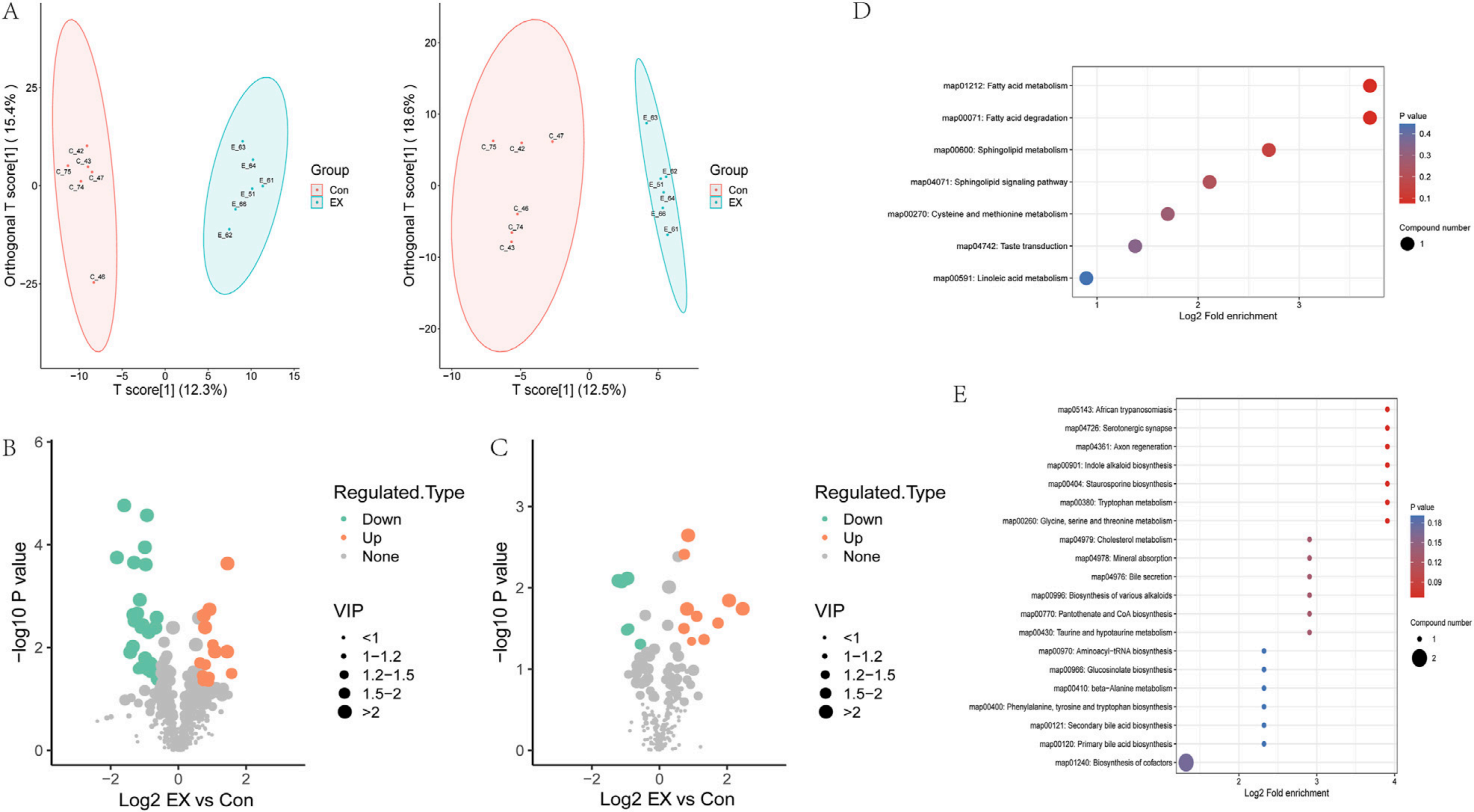
Effects of moderate-intensity exercise on metabolite levels in muscle tissue. **(A)** OPLS-DA analysis of the clustering of samples in the metabolomics data (left, positive ion; right, negative ion). The horizontal coordinate represents the prediction component score (T), and the horizontal coordinate direction can show the difference between groups. The ordinate represents the score value of the orthogonal component (To), and the ordinate direction can show the difference within the group. Percentages represent how well the components explain the dataset. **(B)** Volcano map of differential metabolites in the positive ion. **(C)** Volcano map of differential metabolites in the negative ion. **(D)** KEGG analysis of differential metabolite pathways in the positive ion. **(E)** KEGG analysis of differential metabolic pathways in the negative ion.

Untargeted metabolomics analyzed the changes in metabolite levels in muscle tissue caused by moderate-intensity exercise. Orthogonal partial least squares discriminant analysis (OPLS-DA) analyzed the clustering of samples in the metabolomics data. The results showed that Con and EX muscle tissue samples were clearly separated at the level of the overall metabolome (Figure 5A). The VIP of the OPLS-DA model was analyzed from the obtained multivariate analysis, and differential metabolites were screened. The screening criteria were VIP ≥1.0, *p* value < 0.05, and FC > 1.5 as upregulated differential metabolites. FC < 1/1.5 was a significantly downregulated differential metabolite. The results showed that there were 48 differential metabolites in the positive ion mode, of which 17 were upregulated and 31 were downregulated (Figure 5B; Supplementary Figure 3A). These mainly included amino acids and their metabolites, organic acids, and carbohydrates and their metabolites (Supplementary Figure 3B). There were 17 differential metabolites in the negative ion model, of which 11 were upregulated and six were downregulated (Figure 5C; Supplementary Figure 3C), mainly including amino acids and their metabolites, organic acids, and carbohydrates and their metabolites (Supplementary Figure 3D). KEGG pathway enrichment analyzed for differential metabolites, showed that the pathways enriched for positive ions included the sphingolipid metabolism and sphingolipid signaling pathway involved in sphingosine, Cysteine and methionine metabolism pathways are involved in L-cysteine-glutathione, and fatty acid metabolism and degradation pathways are involved in palmitoyl-L-carnitine (Figure 5D). The top 20 pathways enriched for negative ions include African trypanosomiasis, serotonergic synapse, axon regeneration, indole alkaloid biosynthesis, staurosporine biosynthesis, tryptophan metabolism, glycine, serine and threonine metabolism pathways regulated by L-tryptophan, cholesterol metabolism pathway involved in Taurocholic, biosynthesis of cofactors pathway involved in pantothenic acid and L-tryptophan (Figure 5E).

### 3.6 Correlation analysis between lactated modified proteins and differential metabolites

**FIGURE 6:**
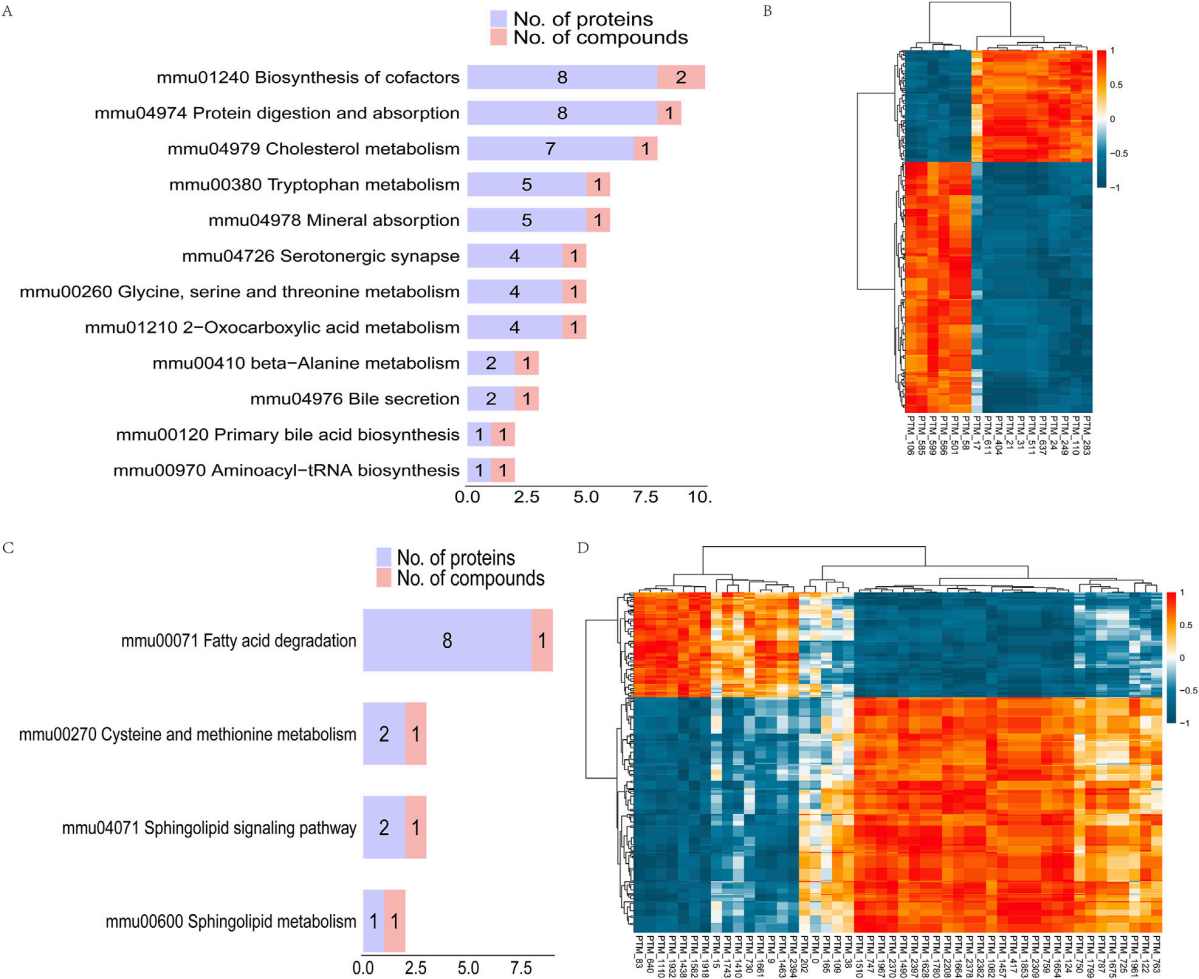
Correlation analysis between lactated modified proteins and differential metabolites. **(A)** KEGG pathway map: pathway in which both differential proteins and negative differential metabolites exist. **(B)** Heat maps of correlation coefficients of differentially expressed proteins and negative differentially expressed metabolites. Red indicates positive correlation and blue indicates negative correlation. The redder the color, the stronger the positive correlation; the bluer the color, the stronger the negative correlation. **(C)** KEGG pathway map in which both differential proteins and positive differential metabolites exist. **(D)** Heat maps of correlation coefficients of differentially expressed proteins and positive differentially expressed metabolites. Red indicates positive correlation and blue indicates negative correlation. The redder the color, the stronger the positive correlation; the bluer the color, the stronger the negative correlation.

In order to study the potential relationship between differential proteins and metabolites in the pathway, we screened the common pathways regulated at the protein and metabolite levels. The results showed that 12 common pathways were regulated at the protein and negative metabolite level, with purple representing the number of differential proteins in this pathway and red representing the number of differential metabolites. The differential metabolites L-tryptophan, taurocholic acid, and pantothenic acid were the main related metabolites. The differential proteins Idh3g, Aco2, Idh1 and Aco1 and the differential metabolite l-tryptophan were found to participate in the regulation of the 2-oxocarboxylic acid metabolism pathway (Figure 6A). Spearman’s analysis of the correlation between differential metabolites and differential proteins showed that L-tryptophan had a significant negative correlation with Idh3g, Aco2, Idh1, and Aco1 (Figure 6B). It was also found that other lactylation-modified proteins had a significant correlation with L-tryptophan, taurocholic acid, and pantothenic acid. Tpm1, Myh1, Gatd3, Atp5mg, and Atp5po had a significant positive correlation with L-tryptophan and taurocholic acid, while Hnrnpa1, Fabp4, and Vim had a significant negative correlation with L-tryptophan and taurocholic acid. Interestingly, we found that Tpm1 and pantothenic acid showed a strong positive correlation coefficient of 1. However, Vim and pantothenic acid showed a strong negative correlation coefficient of −1 (Supplementary Table 4). At the same time, there were four pathways regulated in positive metabolism and protein, and metabolites of sphinganine, L-cysteine-glutathione disulfide, and palmitoyl-L-carnitine were involved in the regulation of the four common pathways. Among these, the acetyl-CoA-related proteins Acsl6, Acat2, Acaa2, Gcdh, Acadvl and Echs1, and palmitoyl-L-carnitine jointly participated in the regulation of the fatty acid degradation metabolic pathway. Gnai2 and kng1 jointly participated in the sphingolipid signaling pathway with sphinganine (Figure 6C). Gnai and kng1 showed a significant negative correlation with sphinganine, while Acaa2, Gcdh, and Acadvl showed a significant positive correlation with sphinganine and a weak negative correlation with palmitoyl-L-carnitine (Figure 6D). Meanwhile, lactylation-modified proteins were significantly correlated with sphinganine, and L-cysteine-glutathione disulfide and palmitoyl-L-carnitine were correlated. Tpm1, Aco2, Mybpn, Myh1, Gatd3, Atp5mg, and Atp5po were significantly positively correlated with sphinganine, while Gatd3, Tpm1, and Atp5po were significantly negatively correlated with palmitoyl-L-carnitine. Fabp4 and Vim were significantly negatively correlated with sphinganine, while Vim was significantly positively correlated with palmitoyl-L-carnitine (Supplementary Table 4).

### 3.7 Expression analysis of key genes and proteins related to lactylation in response to moderate-intensity exercise

**FIGURE 7:**
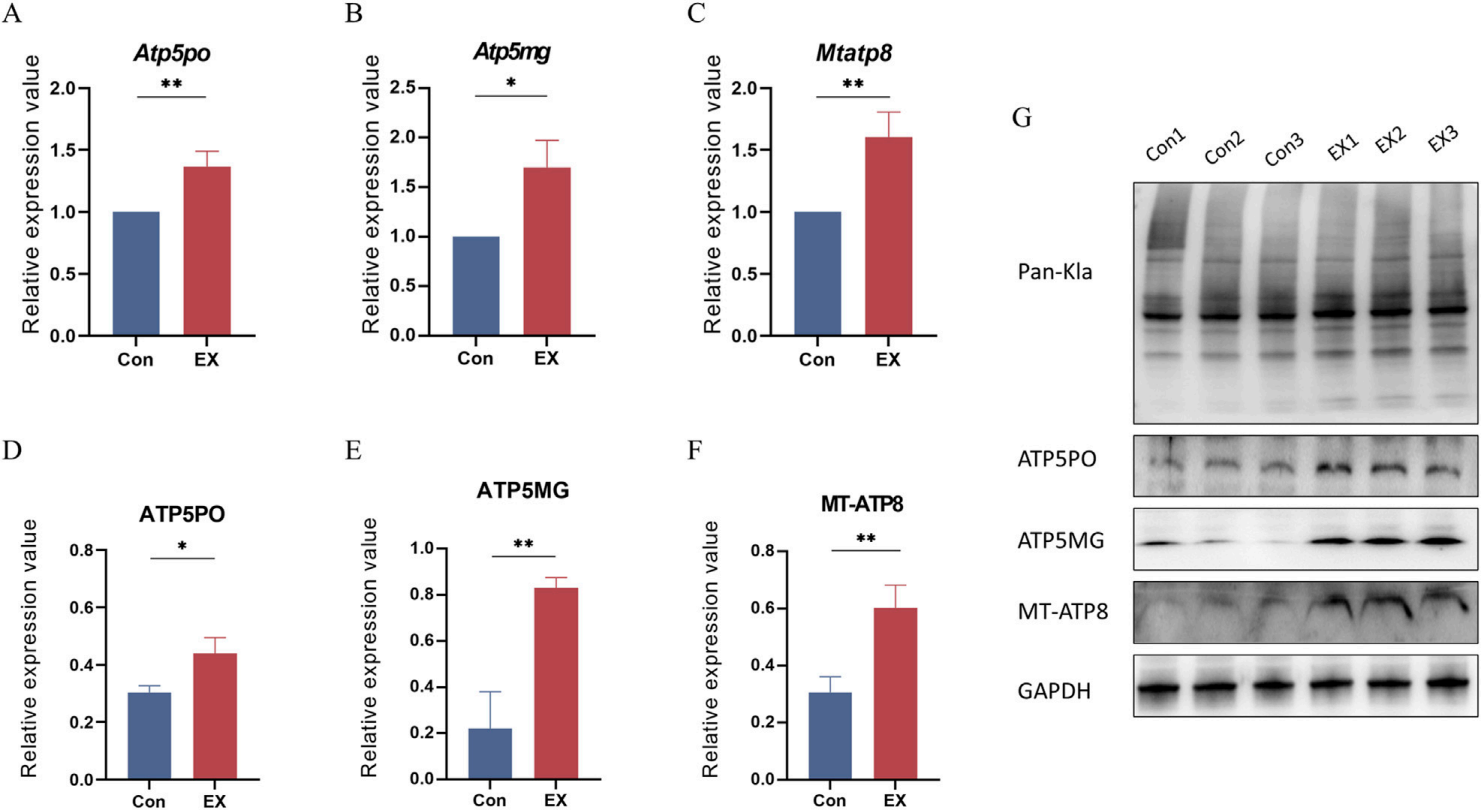
Gene and protein expression levels of Atp5po, Atp5mg, and Mtatp8. **(A–C)**: Relative abundance analysis of Atp5po, Atp5mg, and Mtapt8 mRNA in muscle tissues by RT-qPCR; values expressed as mean ± SEM, n = 3, *p < 0.05, **p < 0.01 for t-test analysis. **(D–F)**: Relative abundance analysis of ATP5PO, ATP5MG, and MT-ATP8 in muscle tissues by Western blot, values expressed as mean ± SEM, n = 3, *p < 0.05, **p < 0.01 for t-test analysis. **(G)**: Bands of Pan-Kla, ATP5PO, ATP5MG, MT-ATP8, and GAPDH proteins.

We further validated the transcriptional and protein expression levels of Mtatp8, Atp5mg, and Atp5po due to their potential involvement in muscle tissue regulation during moderate-intensity exercise. RT-qPCR analysis showed that Mtatp8, Atp5mg, and Atp5po were significantly upregulated (Figures 7A–C). Western blot results further confirmed these transcriptional changes at the protein level (Figures 7D–G). These results highlight the importance of these molecules in exercise-induced lactylation modifications and the regulation of mitochondrial function.

## 4 Discussion

Long-term regular physical activity is beneficial for maintaining and improving cardiovascular, musculoskeletal, and metabolic health while increasing total muscle mass, strength, and explosive power (Ding et al., 2020; Miko et al., 2020). Large amounts of lactic acid accumulate in muscle tissue during exercise, and studies have shown that lactic acid may serve as a metabolic regulator and participate in energy metabolism in muscle cells (Zhou et al., 2022). Lactate is an important carbon-containing metabolite of the cellular glycolysis pathway, and a simple cellular energy substance and metabolite. Zhang et al. (2019) first identified lactate-mediated protein post-translational modification type lactylation (Kla), which plays an important regulatory role in immune cell and cancer metabolism. Studies have shown that protein lactylation modification is an important way for lactate to function, and is involved in important life activities such as uterine remodeling, tumor proliferation, nervous system regulation, and metabolic regulation (Li et al., 2020; Hagihara et al., 2021; Yang et al., 2022). However, the effect of moderate-intensity exercise on overall lactylation in muscle tissue is unknown. We trained mice at moderate intensity and analyzed the changes in overall lactate levels in mouse muscle tissue, finding that 915 lactylation modification sites were identified in 219 quantified proteins, of which 159 lactylation sites in 78 proteins were differentially regulated by moderate-intensity exercise. These results suggest that exercise may be a major regulator of muscle protein lactylation.

We found that a large number of proteins affected by lactylation modification were mainly distributed in different subcellular compartments and not only in cytoplasmic regions. Studies have shown that protein lactylation could link cell metabolism with epigenetic regulation and participate in metabolic reprogramming, glycolytic metabolism, oxidative phosphorylation, and other metabolic pathways (Chen et al., 2022; Fan et al., 2023). Our enrichment analysis showed that the lactated proteins Tpm1 and Myh1 were mainly involved in biological processes such as cardiac muscle contraction and blood circulation, while Myh1, Atp5mg, and Atp5po exhibited ATP hydrolysis activity. Mtatp8, and Atp5po are involved in biological processes such as mitochondrial transmembrane transport and Mtatp8, Atp5mg and Atp5po participate in oxidative phosphorylation and thermogenesis pathways. Furthermore, qPCR and Western blot results also showed a significant increase in transcriptional and protein expression levels in the EX group. Our findings suggest that exercise may affect muscle contraction and circulatory function by altering the lactylation levels of proteins associated with mitochondrial metabolism.

Oxidative phosphorylation (OXPHOS) consumes oxygen to produce ATP. OXPHOS is upregulated in muscle cells to increase energy supply during exercise (Mao et al., 2024). This process of ATP production is to some extent achieved through the conversion of OXPHOS to lactic acid oxidation (Brooks, 2018). The specific mechanisms of oxygen consumption by OXPHOS and the hypoxic conditions produced by exercise have remained elusive (Glancy et al., 2015). A recent study found that lactate levels and mitochondrial oxygen levels are most sensitive to exercise. Muscle cells are sensitive to these two exercise-related indicators at the same time through lactylation and control the production of ATP to control the intensity of exercise and protect the body from excessive exercise injury. Lactylation inhibits the energy metabolism and the level of lactylation modification, which can effectively improve the exercise endurance of mice (Mao et al., 2024). Our results showed that the lactylation levels of Mtatp8, Atp5mg, and Atp5po proteins that are involved in the regulation of mitochondrial transmembrane transport and oxidative phosphorylation in EX are significantly reduced, but the protein levels of Mtatp8, Atp5mg, and Atp5po are significantly increased . Our study further clarified that the reduction of lactate levels induced by exercise is conducive to the regulation of the oxidative phosphorylation pathway and thereby more effectively controls ATP production.

We also explored the effects of moderate-intensity exercise on lactated proteins based on transcriptional and metabolic levels and found that eight lactated proteins (Aco2, Fabp, Hnrnpa1, Mpc2, Mybph, Myh1, Tpm1, and Vim) were quantified at the transcriptional level. Protein and transcriptional levels of Mybph and Tpm1 were significantly upregulated in exercising muscle tissue. Proteomics and metabolomics analysis revealed that Aco2 and differential metabolite L-tryptophan jointly control the 2-oxocarboxylic acid metabolic pathway. Some studies have indicated that tryptophan and its different metabolites have different regulatory effects on animal muscle growth, development, and physiological function (Anaya et al., 2020). Mitochondrial aconitase (Aco2), an enzyme containing iron-sulfur (FeS) clusters, catalyzes the interconversion of citric acid and isocitrate as part of the tricarboxylic acid cycle, producing NADH and FADH2, which drive ATP synthesis via OXPHOS (Chen et al., 2020). The level of Aco2 protein in muscle tissue increases significantly during exercise. The specific role of the oxocarboxylic acid metabolism pathway regulated by Aco2 protein during exercise in muscle tissue needs to be explored.

Our study also found that moderate-intensity exercise had a significant effect on the sphingolipid signaling pathway in muscle tissue. Sphinganine participated in the sphingolipid signaling pathway, which was significantly upregulated in muscle tissue from moderate-intensity training and had a strong positive correlation with Aco2. Sphingolipids accumulate in the skeletal muscle of aging mice, and the inhibition of sphingolipid synthesis prevents age-related decline in muscle mass while enhancing strength and exercise capacity. In the sphingolipid metabolic pathway, the accumulation of dihydroceramide was a key factor interfering with myofibrillar homeostasis (Laurila et al., 2022). The study showed that sphingolipid metabolism plays an important role in regulating the mechanism of muscle growth, differentiation, regeneration, and aging and represents a new strategy for preventing or treating muscle-related diseases (Tan-Chen et al., 2020). Moderate-intensity exercise also has a certain impact on lipid metabolism in muscle tissue, and its specific mechanism can be further explored in future research.

In summary, the results of this project based on transcriptomic, proteomic, lactylation proteomic, and metabolomic analysis have shown that moderate-intensity exercise has an effect on the lactylation level of mice, possibly by reducing the lactylation levels of Mtatp8, Atp5mg, and Atp5po. It also increases the expression of their protein levels, thereby regulating the oxidative phosphorylation pathway and participating in energy metabolism. It was also found that moderate-intensity exercise exerts certain effects on the 2-oxocarboxylic acid metabolism pathway and the sphingolipid signaling pathway in muscle tissue, the specific mechanism of which requires further study.

## Data Availability

The datasets presented in this study can be found in online repositories. Transcriptomics data have been deposited to the Gene Expression Omnibus database with the identifier GSE270280 (https://www.ncbi.nlm.nih.gov/geo/query/acc.cgi?acc=GSE270280). Proteomic data have been deposited to the ProteomeXchange Consortium database with the identifier PXD053276 (https://proteomecentral.proteomexchange.org/cgi/GetDataset?ID= PXD053276). Metabolomics data have been deposited to the EMBL-EBI MetaboLights database with the identifier MTBLS10501 (https://www.ebi.ac.uk/metabolights/MTBLS10501). Further inquiries can be directed to the corresponding authors upon reasonable request.

## Supplementary material

SUPPLEMENTARY FIGURE 1 Overview of lactylation site identification. Total spectra: total and secondary spectra generated by mass spectrometry. Matched spectra: effective spectra matched to theoretical second-order spectra. Peptides: the number of peptide segments were identified and the sequence number of peptide segments from the match result were analyzed. Modified peptides: the number of modified peptides were identified and the sequence number of modified peptides from the match result were analyzed. Identified proteins: the number of proteins that can be resolved by specific peptide segments were identified. Comparable proteins: the number of proteins that can be quantitatively compared, quantified by specific peptides. Identified sites: identified sites. Comparable sites: number of modification bits that can be quantitatively compared.

SUPPLEMENTARY FIGURE 2 Venn diagrams. **(A)**: Quantitative comparison between the transcriptome and the proteome. **(B)**: Comparison of quantitative proteins with lactylation proteins.

SUPPLEMENTARY FIGURE 3 Differential metabolites. **(A)**: Statistical analysis of the amount of the negative differential metabolites. **(B)**: Boxplot analysis of the top-20 negative differential metabolites. **(C)**: Statistical analysis of the amount of the positive differential metabolites. **(D)**: Boxplot analysis of the top-20 positive differential metabolites.
