# Supplementary figures and images for: Multi-omics study on the effect of moderate-intensity exercise on protein lactylation in mouse muscle tissue
Source: Front Cell Dev Biol. 2025 Jan 28;12:1472338. doi: 10.3389/fcell.2024.1472338 (PMC11810897; doi:10.3389/fcell.2024.1472338)

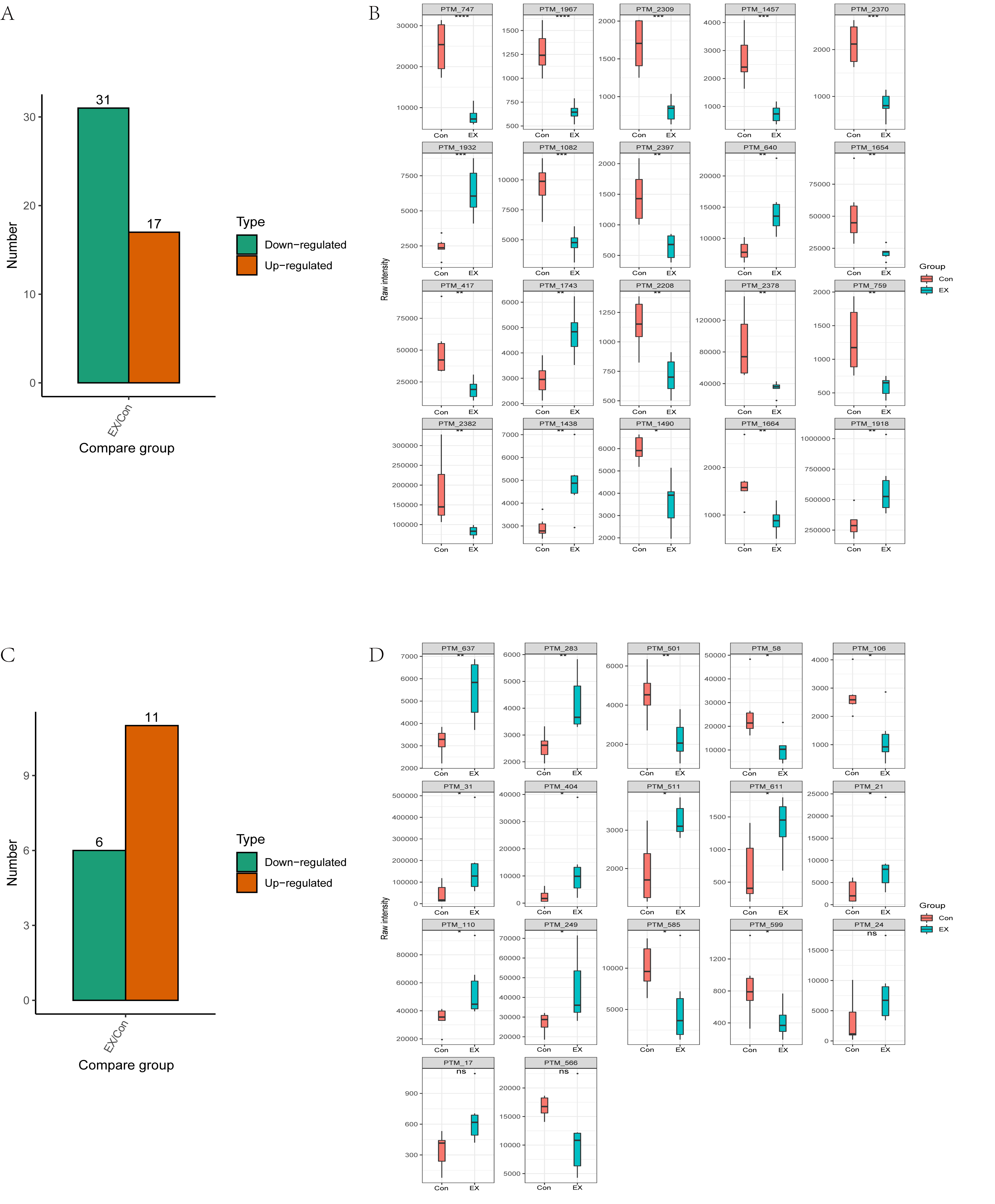

Supplement: Supplementary file 3 [file Image3.tif]

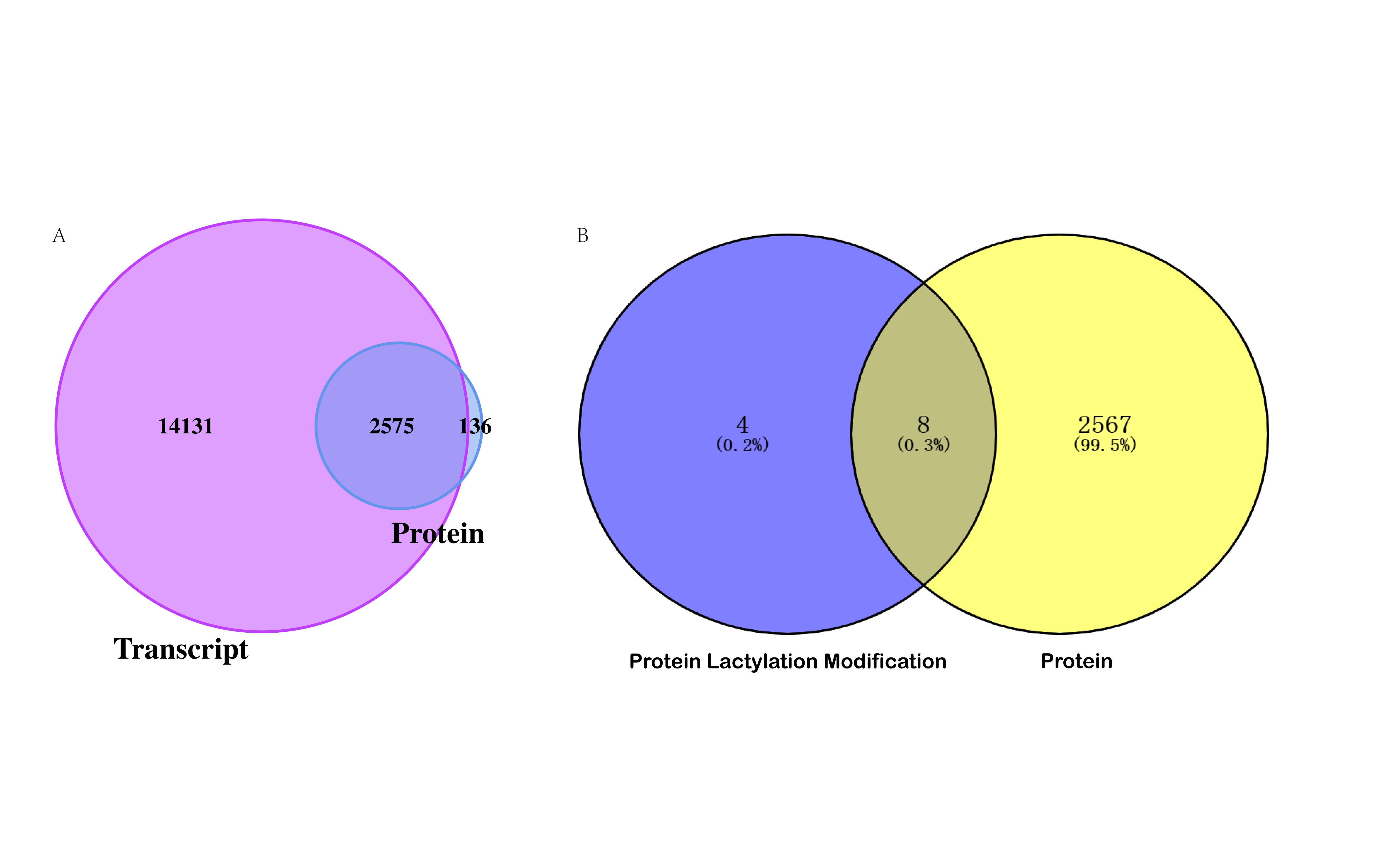

Supplement: Supplementary file 4 [file Image2.tif]

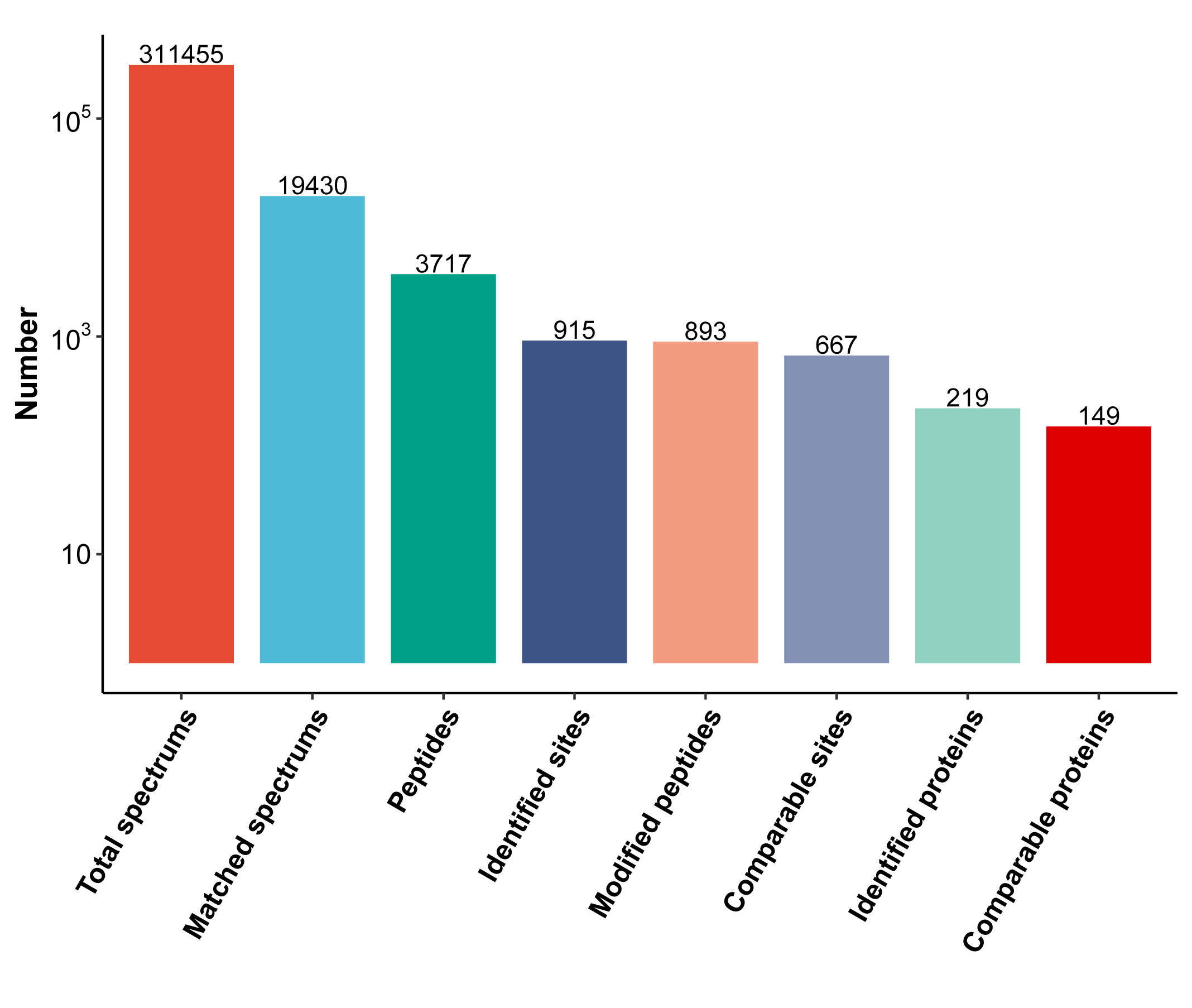

Supplement: Supplementary file 5 [file Image1.tif]
